# Structures of the *E. coli* translating ribosome with SRP and its receptor and with the translocon

**DOI:** 10.1038/ncomms10471

**Published:** 2016-01-25

**Authors:** Ahmad Jomaa, Daniel Boehringer, Marc Leibundgut, Nenad Ban

**Affiliations:** 1 Department of Biology, Institute of Molecular Biology and Biophysics, Otto-Stern-Weg 5, ETH Zurich CH-8093, Switzerland

## Abstract

Co-translational protein targeting to membranes is a universally conserved process. Central steps include cargo recognition by the signal recognition particle and handover to the Sec translocon. Here we present snapshots of key co-translational-targeting complexes solved by cryo-electron microscopy at near-atomic resolution, establishing the molecular contacts between the *Escherichia coli* translating ribosome, the signal recognition particle and the translocon. Our results reveal the conformational changes that regulate the latching of the signal sequence, the release of the heterodimeric domains of the signal recognition particle and its receptor, and the handover of the signal sequence to the translocon. We also observe that the signal recognition particle and the translocon insert-specific structural elements into the ribosomal tunnel to remodel it, possibly to sense nascent chains. Our work provides structural evidence for a conformational state of the signal recognition particle and its receptor primed for translocon binding to the ribosome–nascent chain complex.

## Abstract (web summary)

The co-translational insertion of proteins into membranes requires interaction between a ribosome-bound signal recognition particle (SRP) and a membrane-bound translocon. Here the authors use cryo-EM and single particle reconstructions to obtain a comprehensive view of the co-translational protein targeting process.

## Graphical abstract

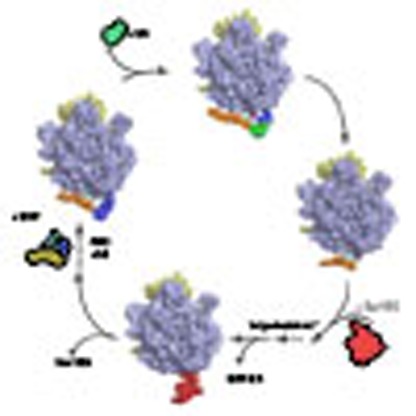

Synthesis of membrane proteins requires that they are co-translationally targeted to the endoplasmic reticulum in eukaryotes and to the cell membrane in bacteria^1^,^2^. This process involves the recruitment of intricate cellular machineries that are conserved throughout all forms of life. This includes a ribonucleoprotein complex termed the signal recognition particle (SRP), the SRP receptor (SR) and the translocation machinery or Sec translocon^3^,^4^,^5^,^6^. Co-translational protein targeting is initiated by the emergence of a hydrophobic N-terminal signal sequence (SS) from a translating ribosome (termed the ribosome–nascent chain complex, RNC). The SRP, consisting of a hairpin-shaped 4.5S RNA (SRP RNA) and Ffh protein (SRP54 homologue in eukaryotes), then binds the RNC^7^,^8^. Ffh is composed of an N-terminal helical and a Ras-like GTPase domain (NG domain)^9^ and methionine-rich M domain that binds to SRP RNA with pico-molar affinity^10^,^11^,^12^. The SRP interaction with the RNC is mediated through both its NG domain and the M domain, which interacts with the SS cargo via a hydrophobic groove sealed by a flanking loop, termed the ‘fingerloop'^10^,^11^. The RNC–SRP complex is then recruited to the membrane by the SR (FtsY in bacteria), which is composed of an Ffh-homologous NG domain^13^ and a largely unstructured A domain that serves to anchor the SR on the membrane and the translocon^14^. Membrane recruitment of the RNC–SRP depends on a guanidine triphosphate (GTP)-mediated heterodimerization of the NG domains of SRP and SR^15^,^16^, resulting in an initial ‘early' state of the SRP–SR complex, in which the NG domain of SR binds to the tetraloop of the SRP RNA hairpin^17^,^18^. Conformational rearrangements in the SRP–SR heterodimer^15^,^16^,^19^ then lead to detachment of the SRP–SR NG dimer from the RNC^8^,^20^,^21^ to form the ‘closed' state^22^. At this stage, the Sec translocon binds to the RNC–SRP–SR complex, and the heterodimerized NG domains of SRP and SR dock at the distal end of the SRP RNA hairpin^23^,^24^,^25^, forming an ‘activated' state where GTP hydrolysis is triggered^26^. Concurrently, the SS is handed over to the Sec translocon, promoting the dissociation of the SRP and the SR once GTP is hydrolyzed^27^. Nascent proteins are then inserted into or translocated across the membrane by the Sec translocon^1^.

Isolated components of the targeting and membrane insertion machinery were previously visualized by X-ray crystallography in different conformational states, including isolated SRP, SRP in complex with SRP RNA and the translocon^10^,^24^,^28^,^29^,^30^,^31^,^32^,^33^,^34^. However, structural information about the binding of the SRP, SR and the translocon to the ribosome in bacteria is only available at lower resolution^21^,^35^,^36^,^37^,^38^, and a comprehensive view on how the SS is recognized by the SRP and then delivered to the translocon is still lacking. Using cryo-electron microscopy, we present structures of the co-translational-targeting pathway intermediates and a complex with Sec translocon at resolutions from 3.4 to 4.3 Å. Our results provide the structural basis of the interaction of the SRP M domain with the RNC, and we present a newly observed state of the SRP–SR complex in the presence of non-hydrolysable GTP analogue that maintains contacts with both the RNC and SRP RNA. In addition, we observe remodelling of the exit tunnel of the RNC and a direct interaction with the nascent chain by the cytoplasmic loops of the Sec translocon. Our results provide evidence for an active role played by the SRP and the Sec translocon on the RNC during cargo recognition and handover.

## Results

### Overview of the co-translational protein-targeting complexes

To obtain high-resolution structures of co-translational-targeting complexes using cryo-electron microscopy, we utilized a well-characterized system to produce SecM-stalled RNC^39^ bearing a modified SS of the PhoA protein, which contains nine leucines and one alanine (1A9L)^40^,^41^,^42^ (Methods). This SS construct exhibits strong binding affinity for both SRP and SRP–SR complexes and has been demonstrated to direct efficient co-translational protein targeting^40^. This approach in combination with the latest cryo-electron microscopy technology enabled us to reconstruct structures of several complexes involved in co-translational targeting and membrane insertion at near-atomic resolution and perform refinement of the atomic coordinates of our models.

### Molecular basis of the RNC–SRP interactions

**Figure 1:**
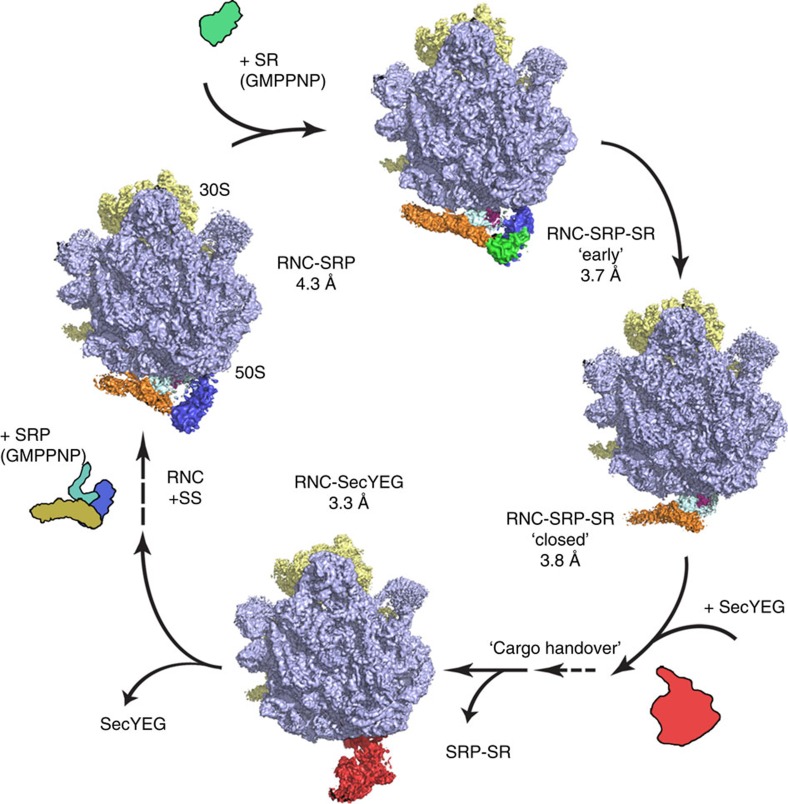
Overview of the visualized co-translational protein-targeting complexes. Structures of the RNC–SRP, RNC–SRP–SR ‘early' and ‘closed' states, and the RNC–SecYEG protein-targeting complexes resolved to near-atomic resolution and placed in sequential order. SRP and SR electron microscopy densities were locally filtered to 6 Å.

**Figure 2:**
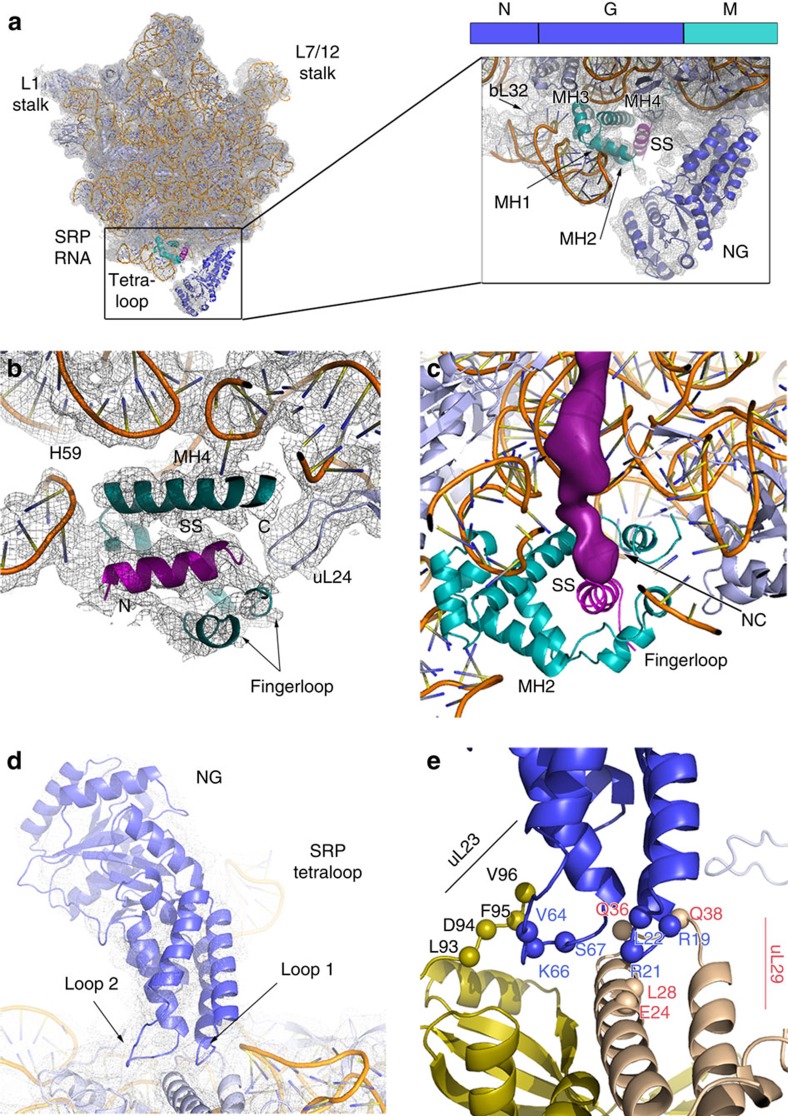
Atomic model of the RNC-bound SRP M and NG domains. (**a**) Cartoon representation of the RNC-bound SRP M and NG domains with overlaid electron microscopy density filtered to 4.3 Å resolution. The electron microscopy density in the zoomed snapshot was locally filtered to 6 Å. NG and M domain are coloured blue and teal; RNA, ribosomal proteins and SS are coloured orange, light blue and magenta, respectively. (**b**) Cross-section of the SS-bound M domain hydrophobic groove with overlaid electron microscopy density filtered to 4.3 Å. (**c**) A view of the polypeptide exit site showing the M domain in the ‘closed' SRP–SR state. The nascent chain (NC) electron microscopy density is displayed as surface (filtered to 6 Å) and coloured in magenta. (**d**) View of the NG domain bound to the RNC, fitted into electron microscopy density. (**e**) Conserved residues, spanning the contact regions, are displayed as spheres. Ribosomal proteins uL23 and uL29 are coloured olive and wheat, respectively.

Our electron microscopy reconstructions reveal two states of the RNC–SRP complex (Supplementary Fig. 1). In one state, SRP RNA and the M domain of the Ffh are ordered, whereas in a second state, we additionally observe the NG domain bound to the RNC. These structures were refined to 3.8- and 4.3 Å resolution, respectively (Fig. 1; Supplementary Fig. 2). The SRP forms three contacts with the ribosomal surface in the vicinity of the nascent polypeptide exit site (Fig. 2a; Supplementary Fig. 3), in agreement with low-resolution studies^36^,^43^. The NG domain binds close to ribosomal protein uL29, positioning the G domain in close proximity to the tetraloop of the SRP RNA. The M domain is located in the vicinity of the tunnel exit, while the SRP RNA contacts the ribosomal protein bL32.

The resolution of the M domain approaches the mean resolution of the ribosome (3.8 Å), which allowed us to build and refine the coordinates of four alpha helices (MH1, MH2, MH3 and MH4) plus the SS (Supplementary Fig. 2c–d; Supplementary Table 1). The M domain interacts almost exclusively with the 23S ribosomal RNA (rRNA; Supplementary Fig. 3a–b), which spans several conserved regions of MH3 and MH4 (Supplementary Fig. 3c–d). The clearly visible density of the SS helix is sandwiched between a hairpin loop of uL24 and the tip of H59 and stacks against MH1 and MH4 (Fig. 2b), which indicates a role for these two ribosomal structural elements in cargo recognition and possibly handover. Although we could not assign the side chains of the SS, which reflects the promiscuity of SS recognition by the M domain, we consider it more likely that the positively charged N terminus of the SS points towards negatively charged H59 rRNA. This orientation is also consistent with the visible path of the nascent chain in the tunnel and the placement of the SS in previous studies (Fig. 2c)^28^,^29^. The density for the C terminus of the M domain, which is disordered in isolated complexes^10^,^12^, can now be visualized. This region forms a short helix (MH5) that seals the SS-binding hydrophobic groove from the bottom. The C terminus further extends into the ribosomal tunnel, where it is in contact with the 23S rRNA, as evidenced by the electron microscopy density contoured at lower threshold (Supplementary Fig. 3a). This location would also allow the C terminus to interact with SSs before their emergence. Consistently, contacts between the C-terminal region of the M domain with the ribosomal tunnel were also observed in the eukaryotic SRP complex^44^.

The interaction areas of the NG domain with the RNC approach the mean resolution of the complex (4.3 Å) and are established through NG loops 1 and 2, which clamp the N-terminal helix of uL29 and contact the C-terminal tail of uL23 (Fig. 2d–e). The interactions include highly conserved residues (Supplementary Figs 4 and 5) mediating both hydrophobic and electrostatic interactions in agreement with previous cross-linking and mutational experiments^7^,^8^,^27^,^45^. Taken together, the interactions of the M and NG domain with the RNC and the positioning of the NG domain in close proximity to the SRP RNA tetraloop indicates that the NG domain is in a state that primes it for the SR binding.

### Architecture of the RNC–SRP–SR complexes

Recognition of the RNC–SRP complex by SR on the membrane initiates GTP-dependent conformational rearrangements that lead to a detachment of the SRP–SR NG domain dimer from the RNC. We assembled the RNC–SRP–SR complex in the presence of 5′-guanylyl imidodiphosphate and could reconstruct the structures of two different states, which were refined to 3.8- and 3.7 Å resolution, respectively (Supplementary Figs 6 and 7). In one state, we observed a detachment of the SRP–SR NG dimer, which was identified as the ‘closed' state of the SRP–SR dimer in a recent cryo-electron microscopy study^21^. Concomitantly with the detachment of this dimer, we can now observe a release of the SRP RNA from its contact with the RNC (Fig. 1; Supplementary Fig. 7e–f). The detachment of the distal region of the SRP RNA may occur to accommodate the ‘activated' state of the NG dimers at the RNA distal site^23^,^24^.

**Figure 3:**
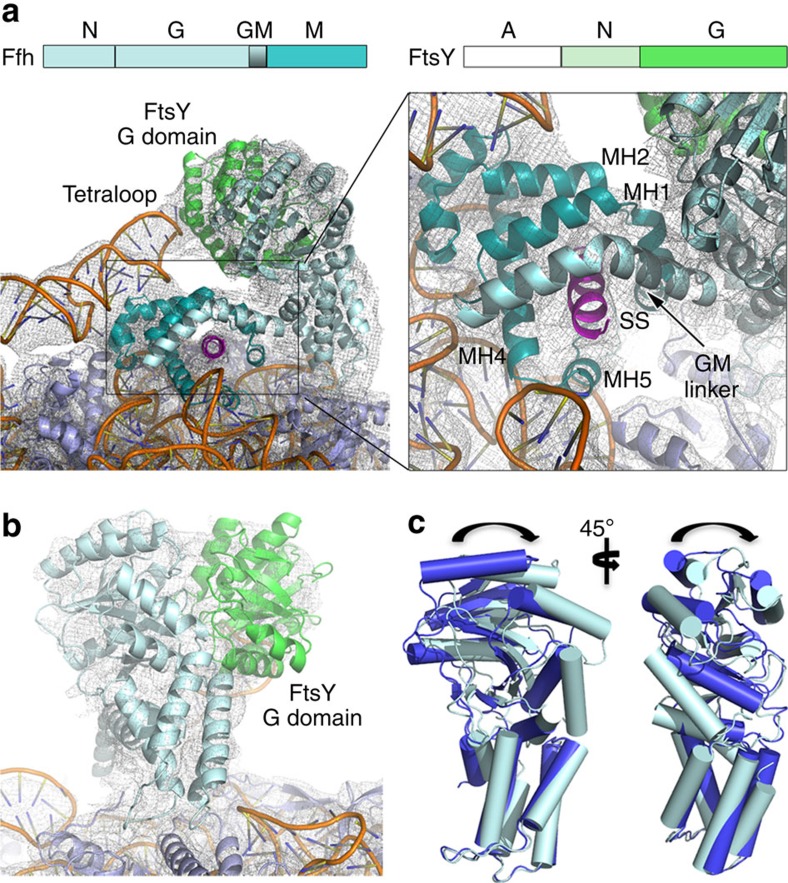
Atomic model of the SRP–SR complex in the ‘early' state. (**a**) Domain structure of the RNC–SRP–SR complex. Representative view of the ‘early' RNC–SRP–SR-targeting complex, depicting the docked SRP–SR NG dimer on the RNC with a magnified snapshot of the M domain. Flexibly disposed A and N domains of FtsY were omitted from the final atomic model. (**b**) Snapshot of the SRP–SR NG domains. The electron microscopy densities of the NG domains and SRP RNA was locally filtered to 6-Å resolution. (**c**) Overlay of the Ffh NG domain in the RNC–SR complex (blue) and the RNC–SRP–SR ‘early' complex (cyan).

In the second state, we identified a conformation of the SRP–SR-targeting complex in which the SR G domain is bound to the SRP RNA tetraloop, whereas the N domain of Ffh is still attached to the RNC at uL23 and uL29 (Fig. 1; Fig. 3a–b). This conformation of the SRP–SR complex was never observed before indicating that it is a short-lived intermediate, and thus we defined it as the ‘early' state of the SRP–SR complex in accordance with previous biochemical data^17^,^18^. The G domains of SRP and SR form a tight complex and when compared with the RNC–SRP complex, the Ffh G domain is rotated relative to the N domain (Fig. 3c; Supplementary Fig. 7c–d). This conformational change could be required to accommodate the SR on the tetraloop. Intriguingly, this state of the Ffh NG domain is distinct from a conformation previously observed in the structures of the isolated Ffh NG domain^9^, the SR–SRP NG dimer^15^,^16^,^46^ and the activated NG dimer bound to the distal site of the SRP RNA^23^,^24^. Furthermore, the GM linker, connecting the NG to M domain, can be observed stacking against the fingerloop and MH1, extending the hydrophobic groove within which the SS is bound (Fig. 3a; Supplementary Fig. 8b). This linker and the preceding sequence exhibit a high degree of conservation (Supplementary Fig. 4), which is consistent with our observation of this region interacting with the SS. This would imply a role for the GM linker in communicating the presence of the SS at the M domain to the attached NG dimer before NG dimer detachment and relocation to the distal site of the SRP RNA.

**Figure 4:**
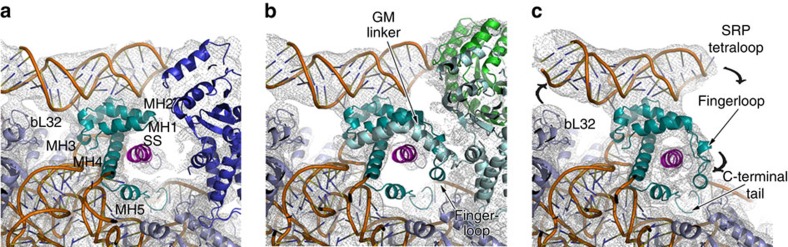
Conformational rearrangements of the M domain during co-translational targeting. (**a**–**c**) Structures of the M domain depicting distinct conformational states in RNC–SRP and RNC–SRP–SR in the ‘early' and ‘closed' states, respectively, with overlaid electron microscopy densities. Curved arrows indicate detachment of the SRP RNA and compaction of the SS hydrophobic groove. The colour scheme is identical to that in Figs 2 and 3.

In addition to the conformational changes in the NG dimer in our visualized complexes, we observe that the M domain of SRP adopts distinct conformations. Notably, in the RNC–SRP complex, the hydrophobic pocket is less well ordered and only weak density for the SS is visible, together with a flexibly disposed fingerloop and GM linker (Fig. 4a; Supplementary Fig. 8a). In contrast, the SS in the ‘early' state of the RNC–SRP–SR-targeting complex exhibits a pronounced α-helical density, packs against MH4 and interacts with MH1 (Fig. 4b), along with a now ordered fingerloop and GM linker (Supplementary Fig. 8b). Fluorescence resonance energy transfer studies have also reported tighter packing of the SS against MH4 in the RNC–SRP–SR compared with the RNC–SRP complex^27^. In the ‘closed' state of the SRP–SR complex, the fingerloop further clamps down onto the SS, compacting hydrophobic groove of the M domain, while the density for the GM linker is now absent (Fig. 4c; Supplementary Fig. 8c). Taken together, these results present further evidence that the conformational states of the M and NG domains are connected and depend on the presence of the SS and the nucleotide state of the SRP–SR GTPase core.

### Atomic model of SecYEG bound to a translating ribosome

**Figure 5:**
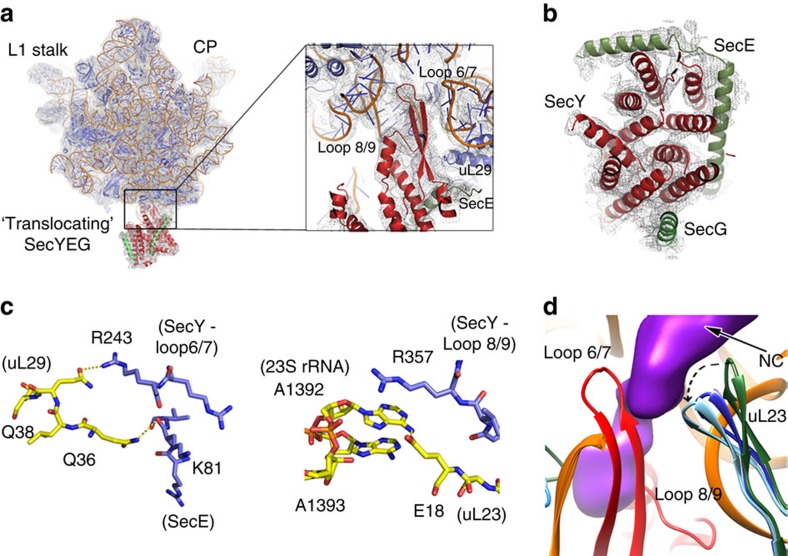
Structure of the ‘translocating' bacterial Sec translocon. (**a**) Atomic model of the RNC–SecYEG complex with overlaid electron microscopy density of the Sec translocon locally filtered to 4.8 Å. (**b**) Representative density of the TM helices of the translocon. (**c**) Contacts between the Sec translocon and the RNC (uL23 and uL29). (**d**) Close-up view of loops 6/7 and 8/9 (red) and the conformation of the hairpin loop of uL23 in the 70S (green), RNC–1A9L and RNC–SRP (orange), and RNC–SecYEG (cyan).

The detachment of the SRP–SR from the tetraloop and docking to the distal SRP RNA site exposes the Sec translocon-binding site on the RNC. We have determined the structure of the RNC in complex with the ‘translocating' *Escherichia coli* Sec translocon (SecYEG) at 3.3 Å (Supplementary Figs 9 and 10a), allowing us to directly visualize the next step in the pathway during co-translational targeting of proteins to the membranes (Fig. 1). Our structure provides a substantial improvement from previous low-resolution cryo-electron microscopy reconstructions^35^,^37^ allowing us to visualize atomic details of the contact points with the RNC and resolve transmembrane helices within the less well-ordered micelle (Fig. 5a–c; Supplementary Fig. 10c). In particular, our structure of the translocating SecYEG reveals a displacement of the plug helix of the aqueous channel in comparison with its position in the structure of the idle translocon (Supplementary Fig. 10d)^31^. In addition, we now observe widening of the lateral gate indicating that the RNC-bound translocon adopts an open conformation (Supplementary Fig. 10e). The contacts with the RNC, including the cytosolic loops of the translocon (loops 6/7 and loop 8/9), can now be visualized at atomic level (∼3.4 Å) and were therefore built *de novo* (Supplementary Fig. 10a–b; Supplementary Table 1). In contrast to the eukaryotic Sec translocon^47^, loop 6/7 of SecY is further inserted into the exit tunnel of the ribosome and sandwiches the nascent chain together with a hairpin loop of uL23 (Fig. 5d; Supplementary Fig. 11a). In particular, this hairpin loop undergoes a shift compared with its position in a recent high-resolution X-ray structure of a non-translating ribosome^48^ and in our RNC–SRP complexes. Loop 8/9 is inserted between rRNA helices H50, and H53, where a contact is established through stacking of residue R357 onto base A1392 (Fig. 5c), in a position similar to the one recently described for the eukaryotic translocon^47^. In contrast to the eukaryotic translocon, which contacts mainly 28S rRNA, loop 6/7 in the bacterial system interacts almost exclusively with ribosomal protein uL29 (Fig. 5c).

Interestingly, the contact residues on uL29 overlap with the SRP NG domain-binding site on the ribosome (Supplementary Fig. 11b). Previous crosslinking^7^,^8^,^45^ and low-resolution cryo-electron microscopy studies^49^,^50^ suggested that both the translocon and SRP interact with both uL23 and uL29. Our data now show that these two factors interact predominantly with uL29, while crosslinks to uL23 occur due to proximity. Moreover, the nascent chain density in the RNC–SecYEG complex is visualized to the end of the ribosomal exit tunnel, where it contacts hairpin loop 6/7. In addition, a tubular electron microscopy density between TM2, TM7 and TM8 at the lateral gate of the translocon is observed, which could represent a density for the SS (Supplementary Fig. 11a).

## Discussion

**Figure 6:**
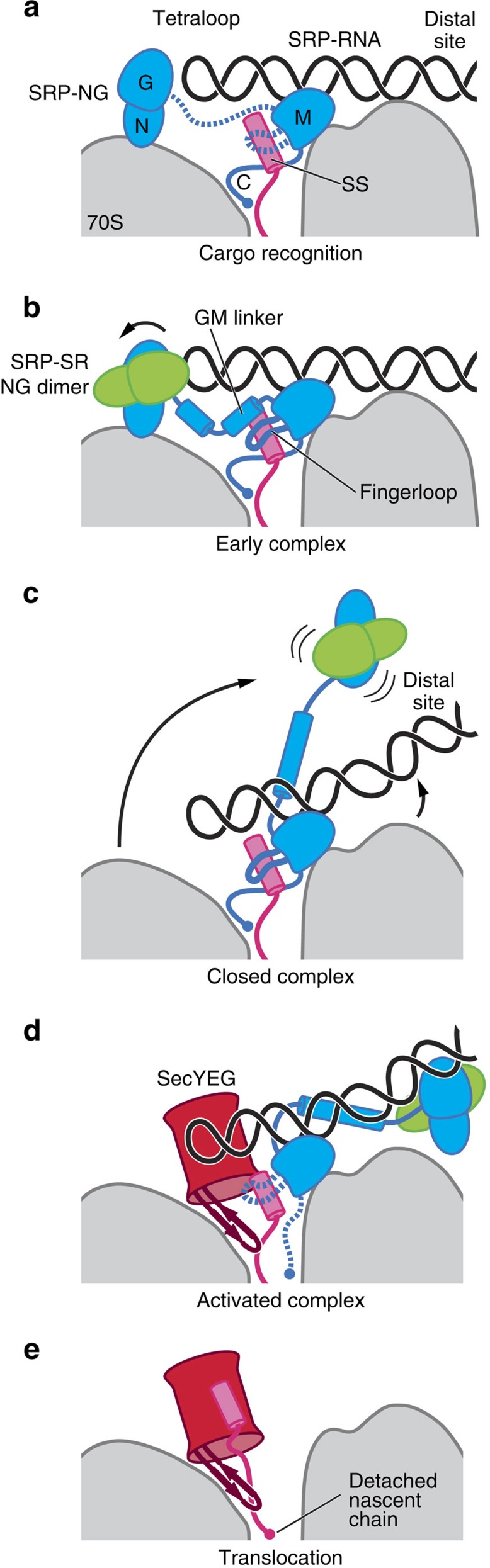
Schematic of the proposed regulatory mechanism of co-translational protein targeting. (**a**–**e**) SRP, SR and SecYEG binding to the RNC based on the structures of the co-translational protein-targeting complexes reported in this study. For details see main text.

Our RNC–SR- and RNC–SRP–SR-targeting complexes imply an interplay between the SS, the fingerloop of the M domain and the GM linker: binding of the SRP to the ribosome involves the formation of interactions between the SRP NG domain, uL23 and uL29, and positioning of the M domain together with the SRP RNA next to the tunnel exit (Fig. 6a). Our data show that the C terminus of the M domain becomes inserted into the ribosomal tunnel, where it is able to interact with the nascent chain. This would imply that the SRP has increased affinity for the translating ribosome even before the SS exits the tunnel, which is consistent with previous biochemical observations^51^,^52^. Higher affinities of SRP binding to the RNC can also be established once the SS emerges from the ribosome tunnel^53^. Recognition of the SS by SRP on the ribosome positions the NG domain and the SRP tetraloop to promote dimerization of the SRP–SR NG domains^38^,^40^. Our results now reveal that the fingerloop remains disordered, presumably due to the conformation of the docked SRP NG domain, which would sterically clash with an ordered fingerloop. Furthermore, our results show that the formation of the ‘early' SRP–SR complex leads to a rotation of the Ffh NG domain and structuring of the GM linker and the fingerloop (Fig. 6b). The ordered GM linker, in the newly observed ‘early' targeting complex, appears to play an important role in binding and positioning the SS. This is in addition to its role in repositioning the NG dimer to the distal region of the SRP RNA at a later stage, where GTP hydrolysis is triggered^24^,^25^. In the ‘closed' SRP–SR complex, we now observe that the fingerloop moves closer to the SS further compacting the hydrophobic groove, whereas the NG domains and the distal region of the SRP RNA detach from the ribosomal surface (Fig. 6c). These results provide evidence for communicating the presence of a SS between the M domain with the NG domain via the fingerloop, which is also corroborated by previous biochemical data^54^.

Our data indicate that the ‘closed' state of the SRP–SR-targeting complex is in a conformation that would allow the translocon to approach the M domain where the SS is bound (Fig. 6d; Supplementary Fig. 11c). Previous Fluorescence resonance energy transfer studies also proposed a simultaneous binding mode of the SRP–SR complex and the Sec translocon at the polypeptide exit tunnel^26^. Intriguingly, the only structural elements from our ribosome-bound SRP that would clash with the translocon are the fingerloop and the C terminus of the M domain. Their position on the ribosome can now be seen occupied by the cytosolic loop 6/7 of the translocon in the RNC–SecYEG complex, suggesting an interplay mechanism between these elements during cargo handover. This is further corroborated by biochemical studies reporting that the deletion of the fingerloop renders the SRP unable to stably engage with the translocation machinery^54^. Once SS handover is complete, the translocon may employ a similar mechanism as the SRP to sense the presence of the nascent chain in the tunnel. In particular, our results imply that the translocon would maintain affinity for the RNC even after the fully synthetized protein detaches from the transfer RNA, by sandwiching the nascent chain between loop 6/7 of the translocon and the hairpin loop of uL23 as long as it is in the tunnel (Fig. 6e).

The structural snapshots of several intermediates of the targeting process presented here provide insights into the molecular mechanism of co-translational protein targeting and membrane protein insertion. Particularly, the complexes resolved in this study track the series of interactions that a particular SS forms during this process, and underscore important roles for the GM linker and the fingerloop of the M domain in coordinating cargo recognition and cargo handover. We also reveal the molecular basis for the interactions between the ribosome, the SRP and the translocon, which allow a better understanding of the spatial rearrangements of these factors on the surface of the ribosome. Finally, our data depict remodelling of the exit site of the ribosomal tunnel, induced by the binding of the Sec translocon and of the SRP, possibly as a nascent chain-sensing mechanism.

## Methods

### Protein expression and purification

The plasmids pET24aFfh and puC19Ffs^38^,^43^ were co-transformed into *E. coli* strain BL21Star(DE3) (Invitrogen) and the cells were grown in LB media at 37 °C. Cultures were induced at an OD_600 nm_ of 0.6 with 1 mM isopropyl-β-D-thiogalactoside for 3 h and broken using a French press in buffer A (50 mM HEPES-KOH, 100 mM KCl, 10 mM MgCl_2_, 1 mM TCEP, pH 8.0). The cleared lysate was purified on His-Trap, MonoQ and S200 columns (GE Healthcare). The presence of the protein component, Ffh, and the SRP RNA, Ffs, was verified using double stained 12% SDS–polyacrylamide gel electrophoresisgels with two drops of 0.1% ethidium bromide added, followed by Comassie brilliant blue. The buffer of purified SRP was exchanged to buffer B (50 mM HEPES-KOH, 100 mM KCl, 10 mM MgCl_2_, 5% glycerol, pH 7.3), and flash frozen and stored at −80 °C until further use. FtsY was expressed from a pET24aFtsY vector, purified and stored in buffer B in a similar manner as described above. The plasmid pTrc99a_SecYEG was transformed in BL21C43(DE3) and the cells were grown at 30 °C in TB media to an OD_600nm_ of 2.0, followed by induction with 1 mM isopropyl-β-D-thiogalactoside at 18 °C overnight. Cells were passed once through a French Press and the lysate was cleared twice in a 70 Ti rotor (Beckman Coulter) at 15,000 r.p.m. for 5 min. Membranes were solubilized for 1 h in buffer A using a final concentration of 1% n-dodecyl β-D-maltopyranoside (DDM) per 10 mg ml^−1^ of protein. Subsequently, solubilized membranes were clarified at 40,000 r.p.m. in a 70 Ti rotor (Beckman Coulter) for 1 h. The supernatant was transferred to a Dounce homogenizer, resuspended in buffer A (+0.02% DDM) and homogenized using 10 strokes. Purification of the resuspended membranes was carried out using His-Trap, SP Sepharose and S200 columns (GE Healthcare). Purified SecYEG sample was dialysed in buffer B (+0.02% DDM) and flash frozen at −80 °C. All purification procedures were performed at 4 °C unless otherwise stipulated.

### Preparation of RNC complexes

RNCs were generated using an *in vitro* translation system using a membrane-free extract from *E. coli* BL21(DE3)^39^. Messenger RNA containing an N-terminal 3 × Strep-tag followed by an engineered SS (1A9L) based on the first 85 amino acids of the PhoA^40^ protein sequence and a SecM-stalling sequence was produced using an cell-free translation system for 30 min at 37 °C. Stalled RNCs were applied on a 10–40% sucrose gradient and subjected to centrifugation for 18 h at 19,000 r.p.m. and 4 °C in a SW 32 Ti rotor (Beckman Coulter) to remove polysomes. Monosomes were then loaded onto a Strep-Tactin Sepharose column (GE Healthcare) and eluted with 2.5 mM D-desthiobiotin (IBA). Eluted fractions were concentrated by pelleting for 3 h in an MLA80 rotor (Beckman Coulter) at 65,000 r.p.m. and 4 °C. Pellets were resuspended in buffer C (50 mM HEPES-KOH, 100 mM KOAc, 10 mM Mg(OAc)_2_, pH 7.3) and flash frozen at −80 °C.

### Cryo-electron microscopy data acquisition

RNC complexes were incubated at 37 °C for 30 min followed by incubation at 4 °C for 30 min using a 1:3 molar excess of factors (either SRP, SRP and SR or SecYEG) to yield a final concentration of 250 nM in reaction buffer (50 mM HEPES-KOH, 50 mM KOAc, 25 mM Mg(OAc)_2_, 0.02% DDM, 2 mM GMPNP, 5 mM spermidine, 0.5 mM spermine). Samples were applied to Quantifoil grids upon which an additional thin layer of carbon had been previously deposited. Samples were incubated for 60 s, blotted for 10 s using filter paper at 6 °C and 100% relative humidity, and then plunged directly into liquid ethane cooled with liquid nitrogen temperature using a Vitrobot (FEI Company). Data were collected on a Titan Krios cryo-transmission electron microscope (FEI Company) operated at 300 KeV and equipped with a Falcon II direct electron detector. EPU software was used for data collection within a defocus range (−0.8 to −3.2 μm) and at × 101,083 magnification. A total of seven frames were collected for each image with a total dosage of 20 electrons per Å^2^. The movie frames were aligned using DOSEFGPU DRIFTCORR^55^ to correct for beam-induced movement.

### Structures calculations

Three different data sets were collected for RNC–SRP, RNC–SRP–SR and RNC–SecYEG complexes, respectively. Overview images of holes were inspected for poor quality ice and cracks in the carbon. The power spectra of individual frames were then carefully inspected and only images exhibiting Thon rings that extended beyond 5 Å were retained. Contrast transfer function (CTF) was estimated using CTFFIND3 (ref. 56). Particles were selected using the batchboxer from EMAN^57^. An initial round of two-dimensional classification was performed on fourfold binned images using the maximum-likelihood refinement algorithm implemented in RELION^58^ to select for two-dimensional averages exhibiting high-resolution features (Supplementary Figs 1, 6 and 9). A three-dimensional classification approach was the preformed using as a reference an empty 70S ribosome low-pass filtered to 50 Å. This step was used to remove ratcheted ribosomes or ribosomes with weakly bound factors. Twofold binned particle images were then refined against an empty 70S ribosome filtered to 50 Å using RELION. An additional classification step, applying a mask over the bound factor area, was performed using local search and skipping alignments, and limiting the resolution to 25 Å. Masks were calculated using SPIDER^59^. Finally, three-dimensional classes exhibiting strong density corresponding to SRP, SRP–SR or translocon were refined by applying a mask on the 50S subunit and using information to Nyquist frequency in RELION. These final maps were used for model building, validation and refinement.

### Model building, refinement and validation

Models of the RNC–SRP and RNC–SecYEG complexes was built using O^60^,^61^ and COOT^62^. The coordinates were refined using PHENIX^63^ as described previously^64^. The atomic coordinates of the 70S^48^, SRP RNA (domain IV) and the Ffh M domain^65^ from *E. coli* were initially docked as a rigid bodies into the cryo-electron microscopy maps. Homology models for the *E. coli* SecY, E and G were obtained using Phyre2 (ref. 66) using the structures of *T. thermophilus* SecY^33^ and *Methanocaldococcus jannaschii* Sec E and G^31^ as templates. Regions of RNA and proteins not fitting into the electron microscopy densities were manually readjusted, including the SecYEG helices. Due to limited local resolution, the fingerloop and the C-terminal extension of SRP were modelled as unassigned (UNK) residues, and residues in loops 1 and 2 were stripped to the backbone.

The resulting models were subjected to nine cycles of individual B-factor and coordinate refinement against figure of merit (FOM) weighted experimental electron microscopy phases and back-calculated structure factors^64^ using the phased maximum-likelihood (MLHL) target. Information during refinement was limited to the FSC gold standard 0.143 cutoff criteria for each cryo-electron microscopy map (Supplementary Table 1). Good main chain geometry, especially for the less well-resolved areas of electron microscopy density, was maintained by imposing base pair, Ramachandran and secondary structural restraints during coordinate refinement^64^. To prevent over-fitting of the coordinates, the weighting between model geometry and structure factors was screened to produce a model with good geometry and low R-values, resulting in fix_wxc values of 1.0 (RNC–SRP–SR, closed), 1.1 (RNC–SRP, class 3) and 1.2 (RNC–SecYEG). Both RNC–SRP (class 3) and ‘closed' RNC–SRP displayed near-identical conformation of the M domain, and thus were used to produce the atomic model for this region. For validation, the refined coordinates were randomly shifted by 0.5 Å and the B-values were reset. Subsequently, both structures were refined into one data half-set of the data following a similar procedure as described above, and the resulting FSCs of the models were compared with the FSCs calculated for the other half-set (Supplementary Figs 2d and 9a).

To interpret the electron microscopy maps of the RNC–SRP with stable NG domain density and the ‘early' RNC–SRP–SR complex, the refined atomic model of the 50S subunit from the RNC–SRP (class 3) complex was fitted as a rigid body into the electron microscopy densities. To account for the rotation of the SRP on the 50S, the Ffh M domain together with SRP RNA and the SS were fitted as a rigid body, and minor adjustments were applied to the distal end of the SRP RNA helix. Although weak electron microscopy density for the distal end of the RNA was visible, this region was not modelled due to its flexibility. Homology models of the NG domain of Ffh (PDB:1JPJ)^67^ and the NG dimers of the Ffh–FtsY (PDB:2J7P)^46^, derived from *Thermus aquaticus* in the GPPNP state, were docked as rigid bodies, followed by manual readjustment of several helices and loops. These included loop 1 and loop 2 of the Ffh N domain contacting the ribosome, the contact area with the SRP RNA of the FtsY G domain, the Ffh N domain helical bundle, which was in a different orientation relative to the G-domain, and the C-terminal helix of the Ffh G domain. Due to limited local resolution, the fingerloop, the GM linker helix and the C-terminal extension of SRP were modelled as unassigned (UNK) residues, and residues in loops 1 and 2 were stripped to the backbone. The N domain of FtsY was only visible at low resolution, which means that this region was flexibly disposed. To correct the final models for geometry errors and to remove sterical clashes, the rebuilt SRP and ribosomal contact areas were subjected to 100 iterations of geometry minimization using PHENIX, while secondary structure, Ramachandran and base pair restraints were applied.

### Making figures and plots

All figures were produced either using UCSF CHIMERA^68^ or Pymol (The Pymol Molecular Graphics System Version 1.7 Schrödinger, LLC.). Local resolution maps were produced using ResMap^69^.

## Additional information

**Accession codes:** Cryo-EM maps have been deposited in the Electron Microscopy Databank with accession codes EMD-8000, EMD-8001, EMD-8002, EMD-8003 and EMD-8004. The coordinates of the atomic structures of the 50S ribosomal subunit in complex SRP–SR, Sec translocon and SRP have been deposited in the protein databank with PDB codes 5GAD, 5GAE, 5GAF, 5GAG and 5GAH.

**How to cite this article**: Jomaa, A. *et al*. Structures of the *E. coli* translating ribosome with SRP and its receptor and with the translocon. *Nat. Commun.* 7:10471 doi: 10.1038/ncomms10471 (2016).

## Supplementary Material

Supplementary Information Supplementary Figures 1-11, Supplementary Table and Supplementary References
