# Supplementary material for: Structures of the E. coli translating ribosome with SRP and its receptor and with the translocon
Source: Nat Commun. 2016 Jan 25;7:10471. doi: 10.1038/ncomms10471 (PMC4737761; doi:10.1038/ncomms10471)
Supplement: Supplementary Information — Supplementary Figures 1-11, Supplementary Table and Supplementary References [file ncomms10471-s1.pdf]

Structures of the E. coli translating ribosome with SRP and its receptor and with the translocon

Ahmad Jomaa[1], Daniel Boehringer[1], Marc Leibundgut[1] & Nenad Ban[1]

[1]Department of Biology, Institute of Molecular Biology and Biophysics, Otto-Stern-Weg 5, ETH Zurich CH-8093, Switzerland. Correspondence and requests for materials should be addressed to N.B. (email: ban@mol.biol.ethz.ch).

Supplementary Information:

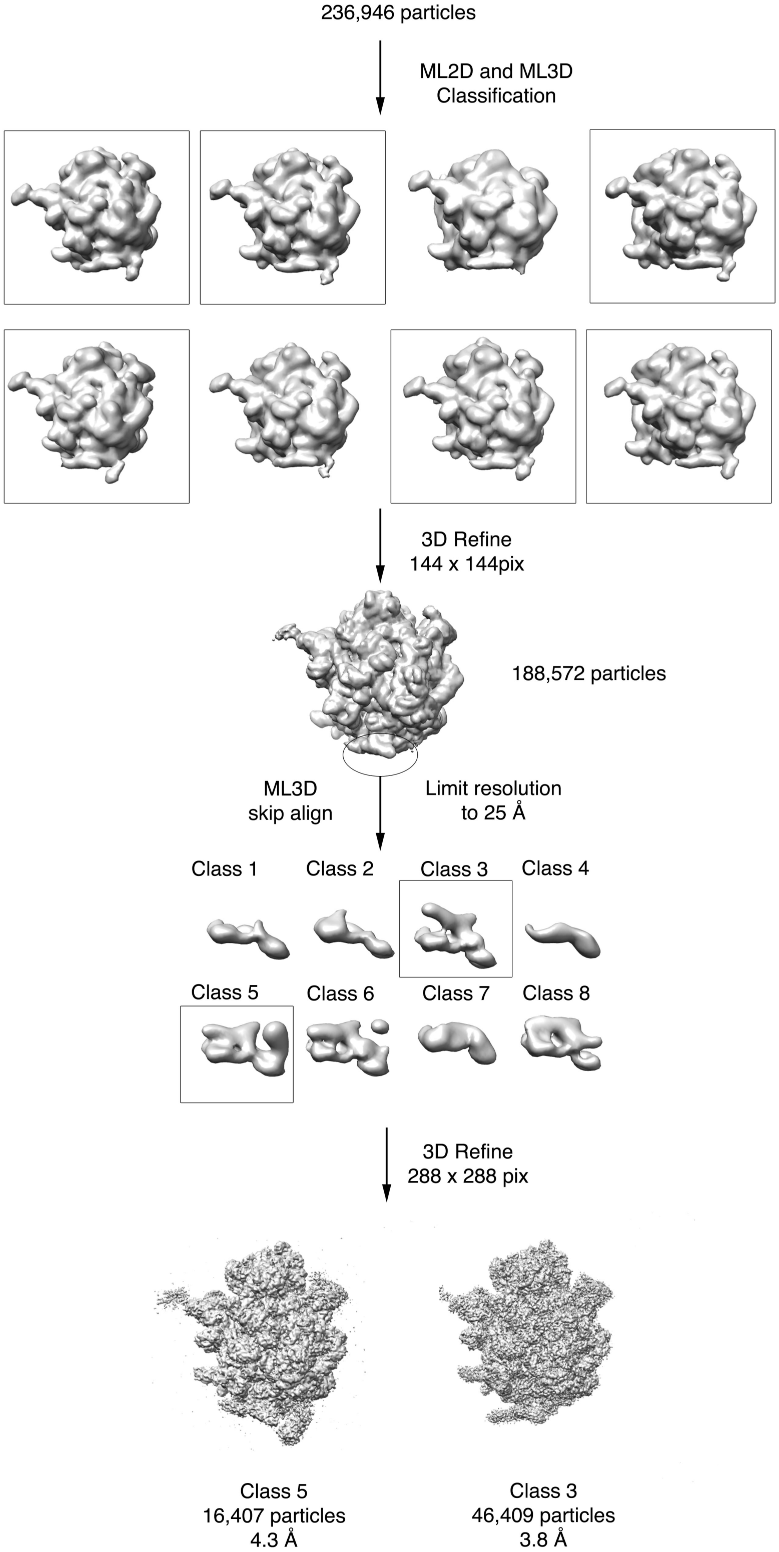

236,946 particles
ML2D and ML3D Classification
3D Refine
144 x 144pix
188,572 particles
ML3D
skip align
Limit resolution to 25 Å
Class 1
Class 2
Class 3
Class 4
Class 5
Class 6
Class 7
Class 8
3D Refine
288 x 288 pix
Class 5
16,407 particles
4.3 Å
Class 3
46,409 particles
3.8 Å

Supplementary Figure 1: Overview of the 2D and 3D classification strategy applied to the RNC-SRP targeting complex. Particles were extracted and binned 4-fold to a box size of 80x80 pixels with a sampling of 5.54 Å/pixel and then exposed to a maximum-likelihood 2D classification approach using RELION to initially remove particles of low quality. Particles were then subjected to 3D classification using a low-pass filter cutoff to 50 Å resolution to an empty *E. coli* 70S ribosome as reference (number of classes=8). Classes were further sorted based on the existence of P- and E-site tRNA and SRP density. Ratcheted ribosomes and those containing weak SRP density were excluded. Remaining classes were combined into one class and refined using the empty 70S *E. coli* ribosome filtered to 50 Å resolution and 2-fold binned images (160x160 pixels). A semi-circular mask was then applied on the SRP area to include the SRP RNA and M and NG domains, and particles were then exposed to a focused 3D classification approach using the 'skip align' command in RELION. Fine angular sampling was performed while the resolution was limited to 25 Å. An empty 70S ribosome filtered to 30 Å as a reference (number of classes=8). Classes displaying density for the SRP RNA and the Ffh NG-domains were further refined separately from full-sized images (320x320 pixels) using information up to Nyquist frequency with a sampling of 1.385 Å/pixel and an empty ribosome filtered to 50 Å as a reference after applying a mask on the 50S subunit. The final resolution and number particles are reported at the bottom of the figure. A gold standard FSC cutoff of 0.143 was used as implemented in RELION.

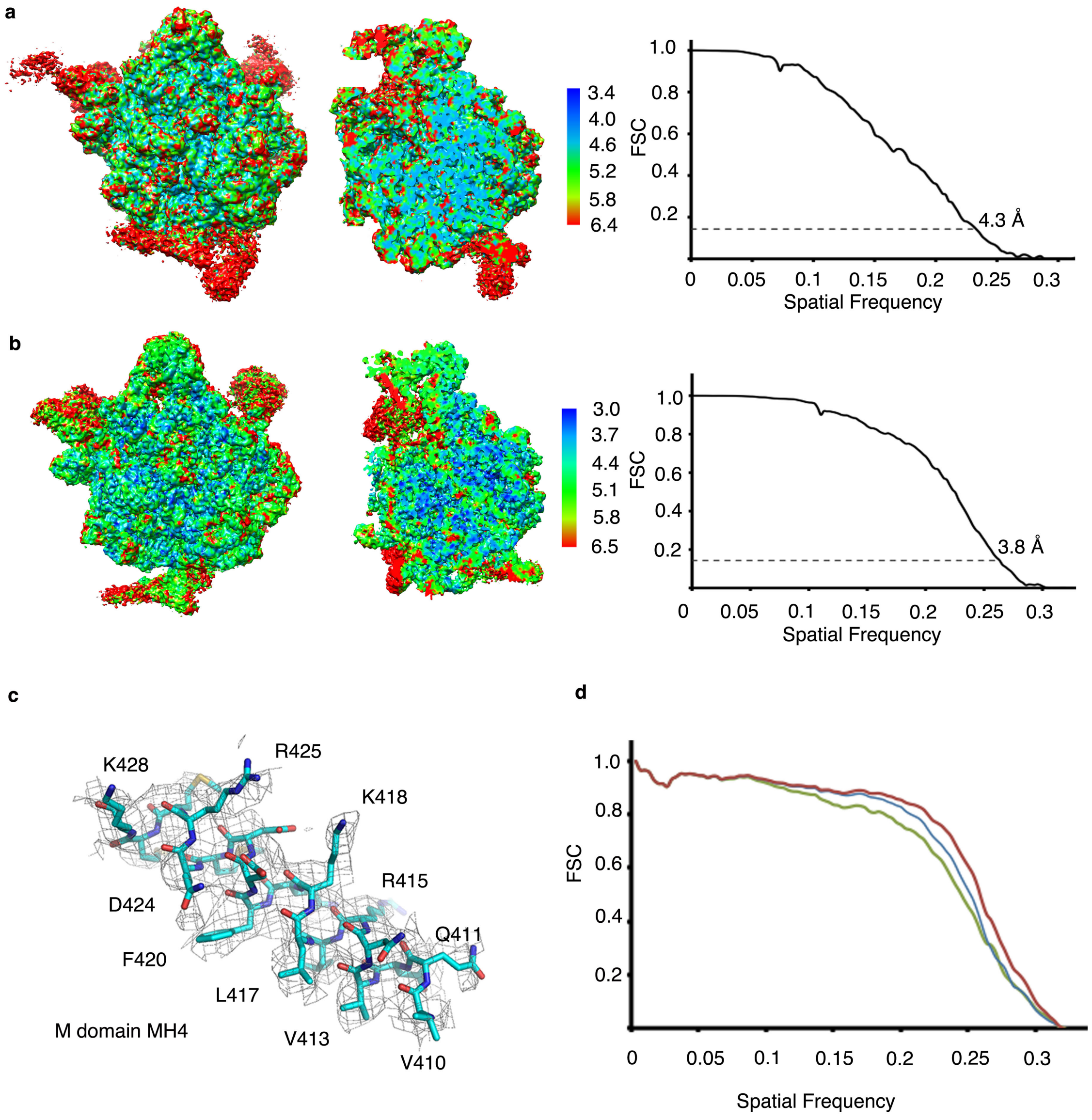

Supplementary Figure 2: Overview of the RNC-SRP complexes. a and **b**, Local resolution and gold standard FSC plots of Class 5 and Class 3 of the RNC-SRP targeting complex, respectively. The FSC cutoff value used was 0.143. **c**, Representative EM density filtered to 3.8 Å with built coordinates of H4 of the M domain of the RNC-SRP targeting complex (Class 3) displayed as sticks. **d**, Refinement weight validation plot for the RNC-SRP refinement. FSC calculated for model versus map for half-dataset 1 (used for refinement) in blue, versus map for half-dataset 2 (not used for refinement) in green, versus full dataset in red. Larger gap for green versus blue plots would indicate over-refinement.

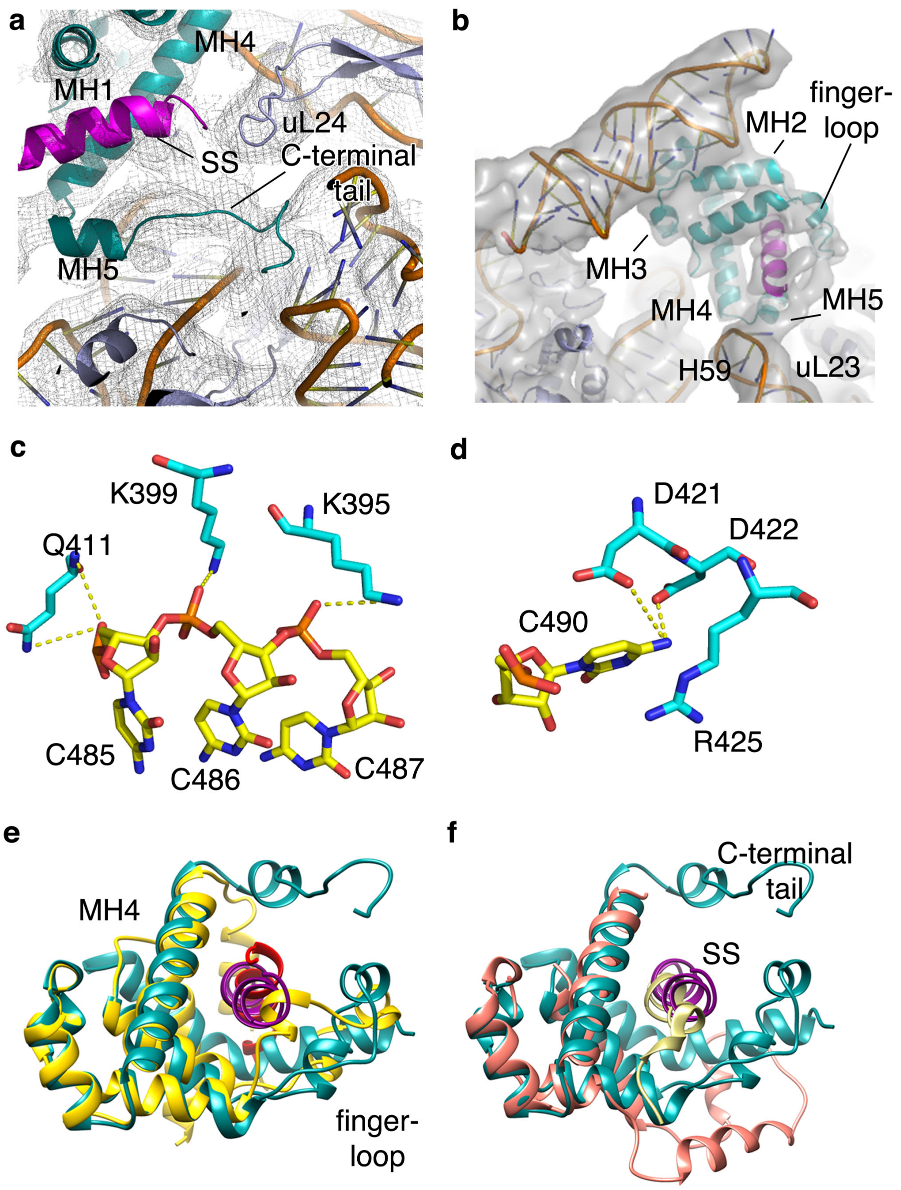

Supplementary Figure 3: Overview of the RNC-bound M domain. a, Cartoon representation of the polypeptide exit site with an overlay EM-density of the RNC-SRP complex locally filtered to 6 Å. **b**, Atomic model of the M domain with an overlay of semi-transparent surface representation of the EM-density obtained from co-translational targeting complexes reported in this study. RNA was colored orange, ribosome protein blue, M domain teal, and SS in magenta. **c** and **d,** Stick representations of the contacts between MH3 and MH4 of the Ffh M domain and the 23S rRNA at the polypeptide exit site of the RNC. **e** and **f**, Overlay of the atomic models of the isolated SS-bound M domain from *Solflubus solfataricus* (PDB:3KL4; yellow, signal sequence; red) and *Methanocaldococcus jannaschii* (PDB: 4XCO; salmon, signal sequence; wheat) and the M domain of RNC-SRP complex from this study.

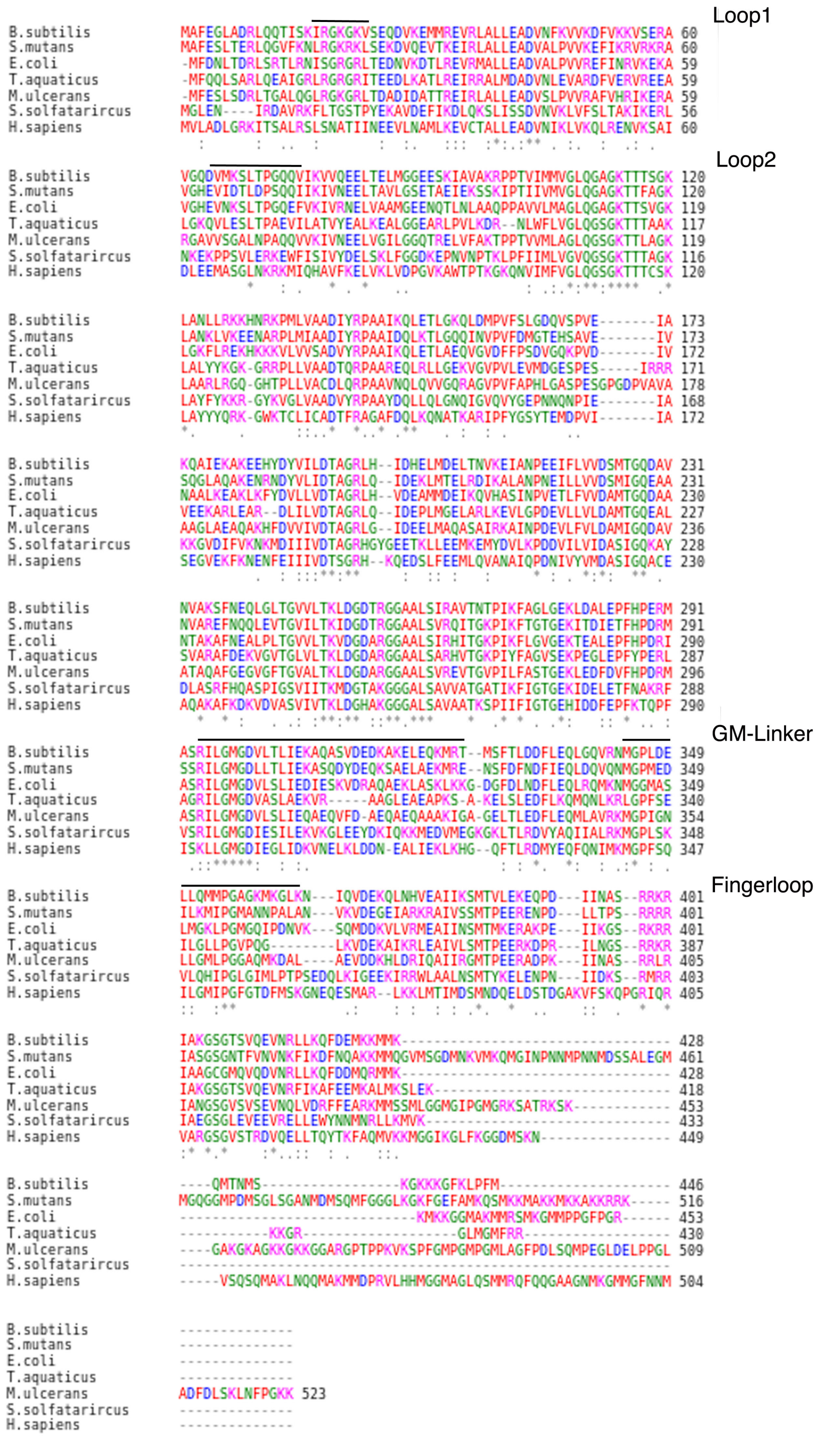

Supplementary Figure 4: Sequence alignment of the Ffh domains from different organisms. Sequences of loops 1 and 2, GM linker, and fingerloop are highlighted. Sequence alignment was produced using the ClustalW WebServer [1,2].

Ribosomal protein uL29

```
E.coli              ----MKAKELR-------EKSVEELNTELLNLLREQFNLR-MQAASGQLQQSHLLKQVRR 48
S.enterica          ----MKAKELR-------EKSVEELNTELLNLLREQFNLR-MQAASGQLQQSHLLKQVRR 48
T.thermophilus      ----MKLSEVRKQLEEARKLSPVELEKLVREKKRELMELR-FQASIGQLSQNHKIRDLKR 55
D.radiodurans       ----MKPSEMR-------NLQATDFAKEIDARKKELMELR-FQAAAGQLAQPHRVRQLRR 48
B.subtilis          ----MKANEIR-------DLTTAEIEQKVKSLKEELFNLR-FQLATGQLENTARIREVRK 48
H.marsimortui       MTV-LHVQEIR-------DMTPAEREAELDDLKTELLNARAVQAAGGAPENPGRIKELRK 52
M.tuberculosis      MAVGVSPGELR-------ELTDEELAERLRESKEELFNLR-FQMATGQLNNNRRLRTVRQ 52
H.sapiens           -MAKIKARDLR-------GKKKEELLKQLDDLKVELSQLRVAKVTGGAASKLSKIRVVRK 52
                        :   ::*            :    :     *  : *  : : *   :   :: :::

E.coli              DVARVKTLLNEKAGA--------------------------------------------- 63
S.enterica          DVARVKTLLTEKAGA--------------------------------------------- 63
T.thermophilus      QIARLLTVLNEKRRQNA------------------------------------------- 72
D.radiodurans       EVAQLNTVKAELARKGEQQ----------------------------------------- 67
B.subtilis          AIARMKTVIRER-EIAANK----------------------------------------- 66
H.marsimortui       AIARIKTIQGEEGDLQENE----------------------------------------- 71
M.tuberculosis      EIARIYTVLRER-ELGLATGPDGKES---------------------------------- 77
H.sapiens           SIARVLTVINQTQKENLRKFYKGKKYKPLDLRPKKTRAMRRRLNKHEENLKTKKQQRKER 112
                     :*:: *:  :
```

Ribosomal protein uL23

```
E.coli              ------MIREERLLKVLRAPHVSEKASTAMEKSNTIVLKVAKDATKAEIKAAVQKLFEVE 54
S.enterica          ------MIREERLLKVLRAPHVSEKASTAMEKTNTIVLKVAKDATKAEIKAAVQKLFEVE 54
T.thermophilus      ---------MKTAYDVILAPVLSEKAYAGFAEG-KYTFWVHPKATKTEIKNAVETAFKVK 50
D.radiodurans       ----------MSHYDILQAPVISEKAYSAMERG-VYSFWVSPKATKTEIKDAIQQAFGVR 49
B.subtilis          ---------MKDPRDVLKRPVITERSADLMTEE-KYTFEVDVRANKTEAKDAVESIFGVK 50
M.tuberculosis      ------MATLADPRDIILAPVISEKSYGLLDDN-VYTFLVRPDSNKTQIKIAVEKIFAVK 53
H.sapiens           PRKSAPRRNKLDHYAIIKFPLTTESAMKKIEDNNTLVFIVDVKANKHQIKQAVKKLYDID 120
H.marsimortui       -----------MSWDVIKHPHVTEKAMNDMDFQNKLQFAVDDRASKGEVADAVEEQYDVT 49
                                   ::  *  :* :   :       : *   :.* :   *::  : :

E.coli              VEVVNTLVVKGKVKRHGQRIGRRSDWKKAYVTLKEGQN-LDFVGGAE-- 100
S.enterica          VEVVNTLVVKGKVKRHGQRIGRRSDWKKAYVTLKEGQN-LDFVGGAE-- 100
T.thermophilus      VVKVNTLHVRGKKKRLGRYLGKRPDRKKAIVQVAPGQK-IEALEGLI-- 96
D.radiodurans       VIGISTMNVPGKRKRVGRFIGQRNDRKKAIVRLAEGQS-IEALAGQA-- 95
B.subtilis          VDKVNIMNYKGKSKRVGRYTGMTSRRRKAIVKLTADSKEIEIFEA---- 95
M.tuberculosis      VASVNTANRQGKRKRTRTGYGKRKSTKRAIVTLAPGSRPIDLFGAPA-- 100
H.sapiens           VAKVNTLIRPD---------GE----KKAYVRLAPDYDALDVANKIGII 156
H.marsimortui       VEQVNTQNTMD---------GE----KKAVVRLSEDDDAQEVASRIGVF 85
                    *  :.     .         *     ::* * :  .    :
```

Hairpin loop

Supplementary Figure 5: Sequence alignments of the uL23 and uL29 from different organisms. The hairpin loop region of uL23, which extends into the tunnel of the ribosome is highlighted.

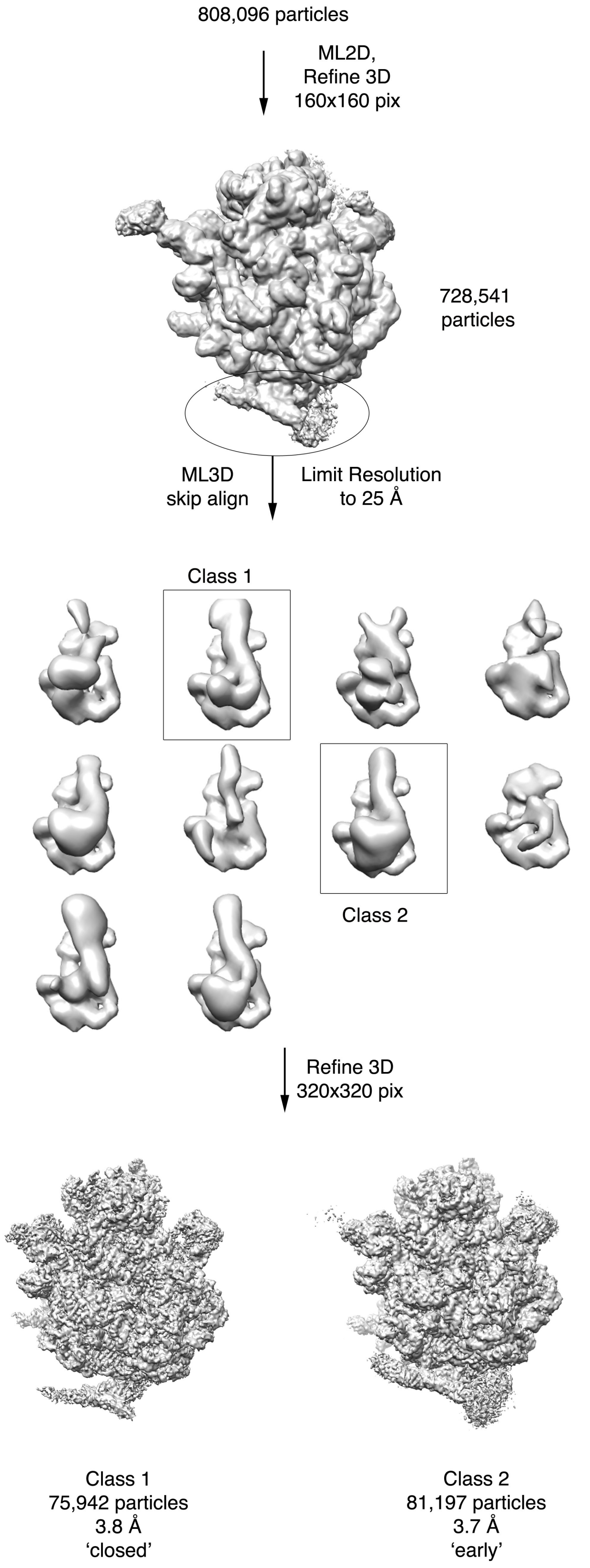
808,096 particles
ML2D,
Refine 3D
160x160 pix
728,541
particles
ML3D
skip align
Limit Resolution
to 25 Å
Class 1
Class 2
Refine 3D
320x320 pix
Class 1
75,942 particles
3.8 Å
'closed'
Class 2
81,197 particles
3.7 Å
'early'

Supplementary Figure 6: An overview of the 2D and 3D classification used for the RNC-SRP-SR targeting complex. An image classification scheme as shown in Extended Data Figure 1 was followed with slight modifications: The initial 3D classification step was skipped and directly applied the focused 3D classification approach using RELION after 2D classification and initial refinements. Two classes displaying stable densities for the SRP-SR NG domains and M domain were selected and refined. Empty ribosomes low-pass filtered to 50 Å and 30 Å were used as references in the refinement and focused 3D classification steps, respectively. Further re-classification of the classes with the density for the NG-dimers did not improve the quality of the maps. Final refinement was done with a mask applied on the 50S subunit to exclude the 30S subunit and to improve the alignments of 50S region.

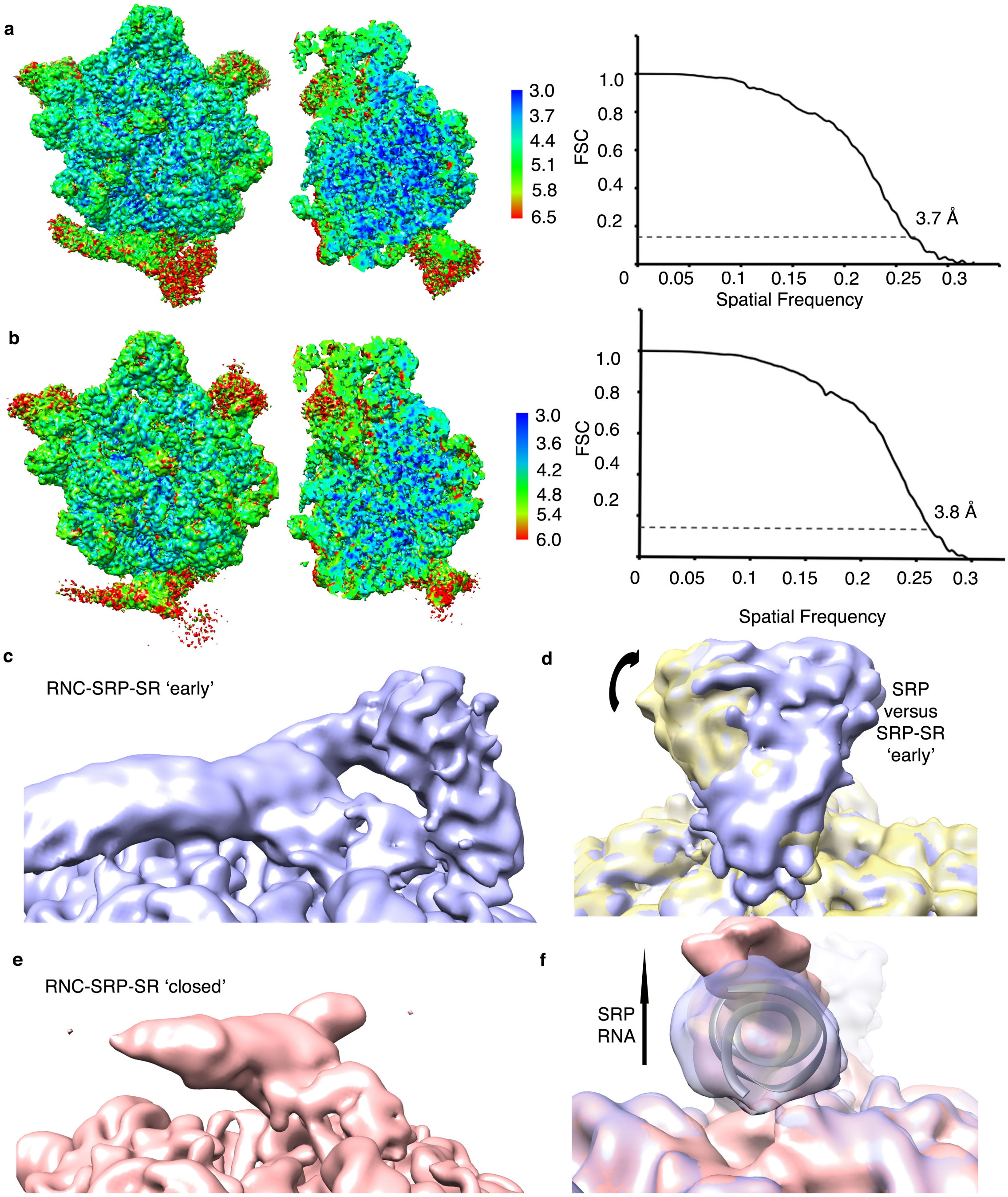

Supplementary Figure 7: Overview of the RNC-SRP-SR conformational states. a and **b**, Local resolution and the FSC gold standard plots of two states of the RNC-SRP targeting complexes. The cutoff criteria used was 0.143. **c** and **d**, Close-up view of the SRP RNA region with visible densities of the NG dimer and M domain. **e**, Overlay-density of the RNC-SRP-SR in the'early' state (purple) and RNC-SRP (yellow). **f**, Overlay-density of the 'early' RNC-SRP-SR (purple) and 'closed' RNC-SRP-SR states (salmon). Cryo-EM maps were filtered to 8 Å.

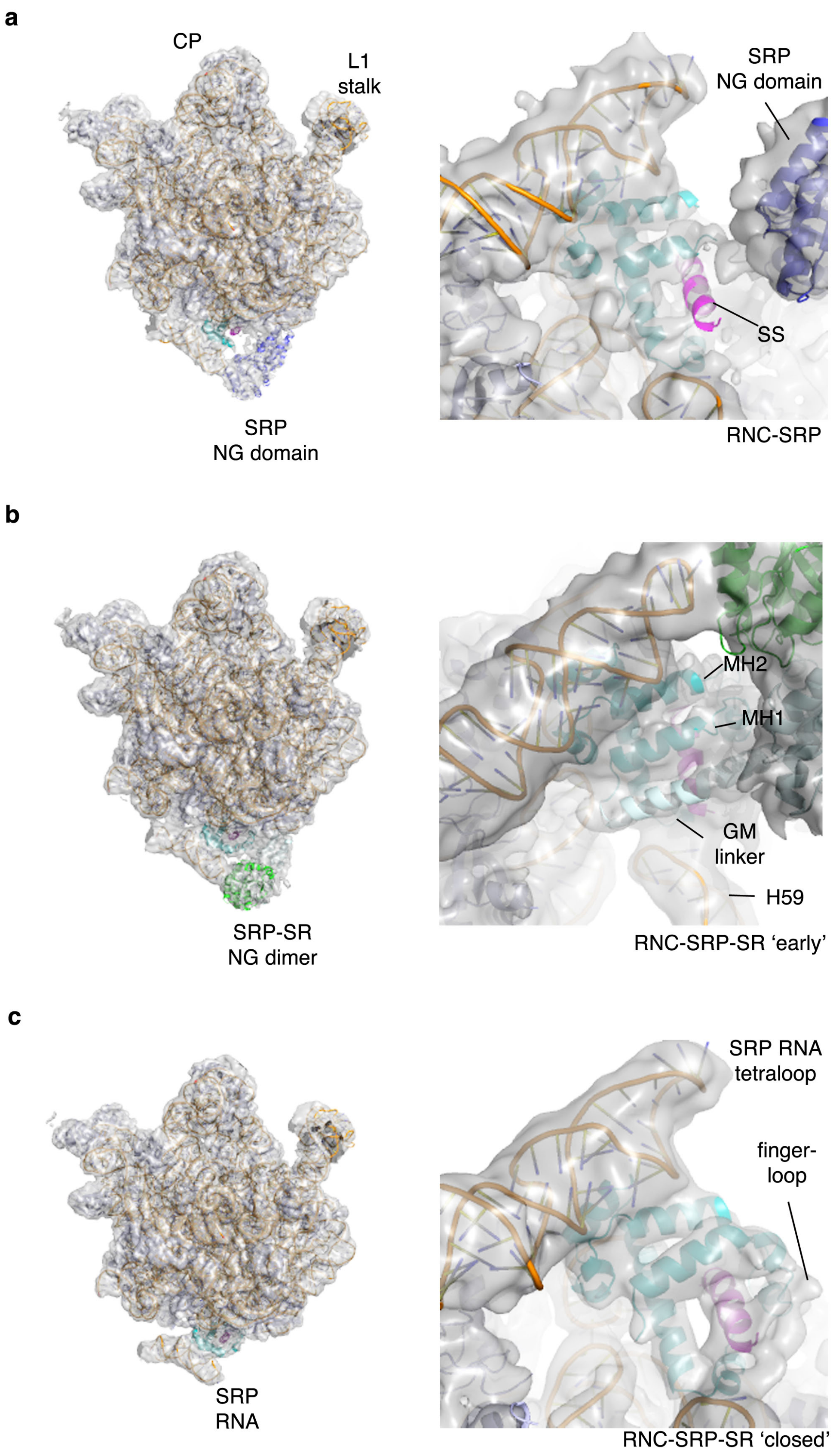
a
CP
L1
stalk
SRP
NG domain
SRP
NG domain
SS
RNC-SRP
b
SRP-SR
NG dimer
MH2
MH1
GM
linker
H59
RNC-SRP-SR 'early'
c
SRP
RNA
SRP RNA
tetraloop
finger-
loop
RNC-SRP-SR 'closed'

Supplementary Figure 8: Overview of the M domain of SRP during protein targeting. (a-c) Atomic models of the RNC-SR, RNC-SRP-SR 'early' and 'closed' complexes with an overlay semi-transparent surface of the corresponding EM-densities. Left panels depict zoomed areas of the SRP M domains. Surface densities were filtered to 6Å resolution. Coloring scheme is identical to the one used in main figures.

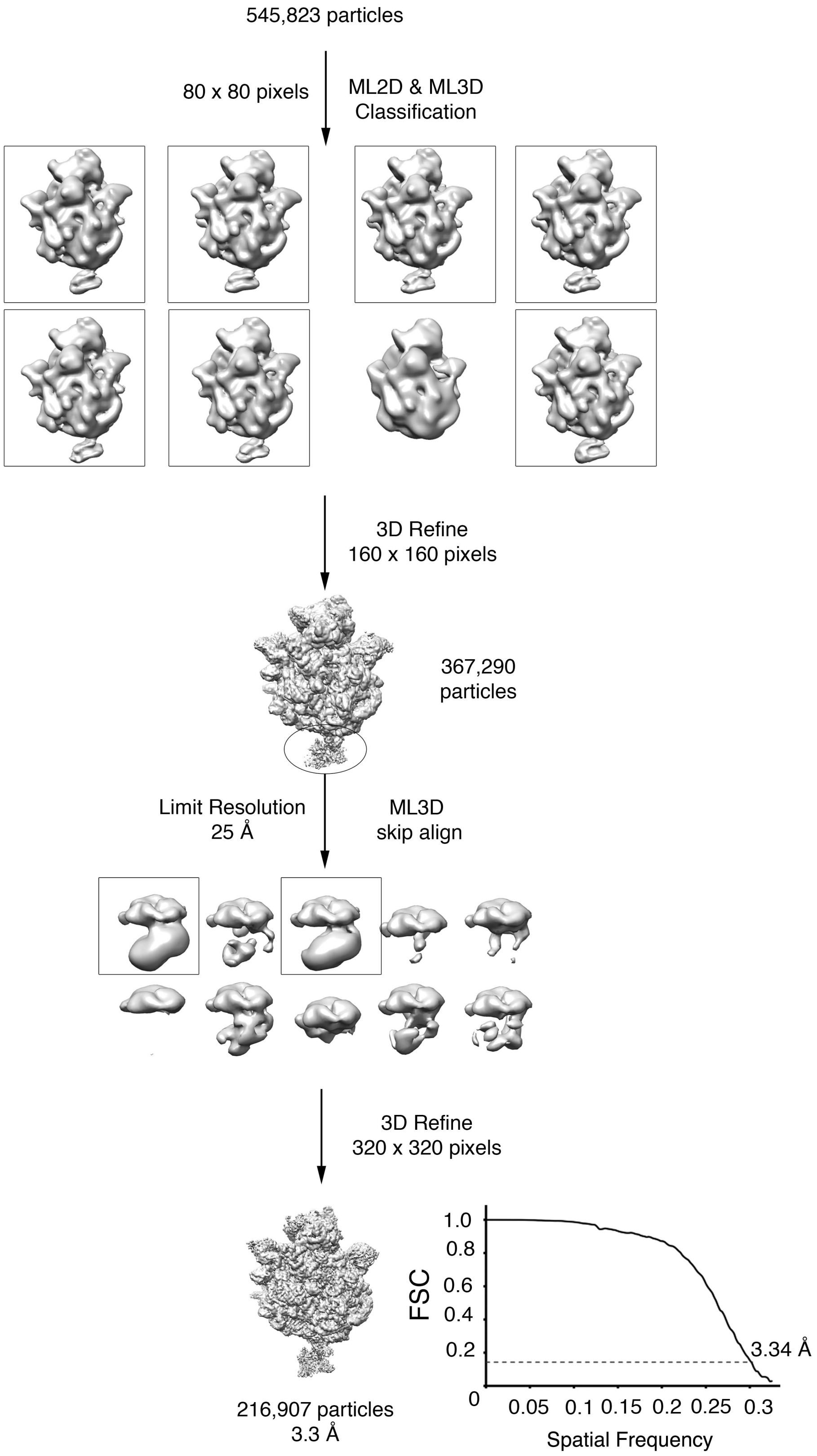
545,823 particles
80 x 80 pixels
ML2D & ML3D
Classification
3D Refine
160 x 160 pixels
367,290
particles
Limit Resolution
25 Å
ML3D
skip align
3D Refine
320 x 320 pixels
216,907 particles
3.3 Å
1.0
0.8
0.6
0.4
0.2
0
FSC
3.34 Å
0.05 0.1 0.15 0.2 0.25 0.3
Spatial Frequency

Supplementary Figure 9: An overview of the 2D and 3D classification approach of the RNC-SecYEG targeting complex. A similar approach was applied as described above in Figures S1 and S6. In brief, binned particles were exposed to 2D then 3D classification to remove particles that do not contain density at the tunnel region. A focused classification approach was then performed using 10 classes, as described earlier. A circular mask was applied around the area where the translocon is bound. Finally, two classes with strong density for the translocon were merged and then refined using full size images. Further reclassification of this subpopulation did not improve the density of the translocon. Focused refinements were carried using an empty 70S ribosome filtered to 50 Å as a reference after applying a mask on the 50S subunit.

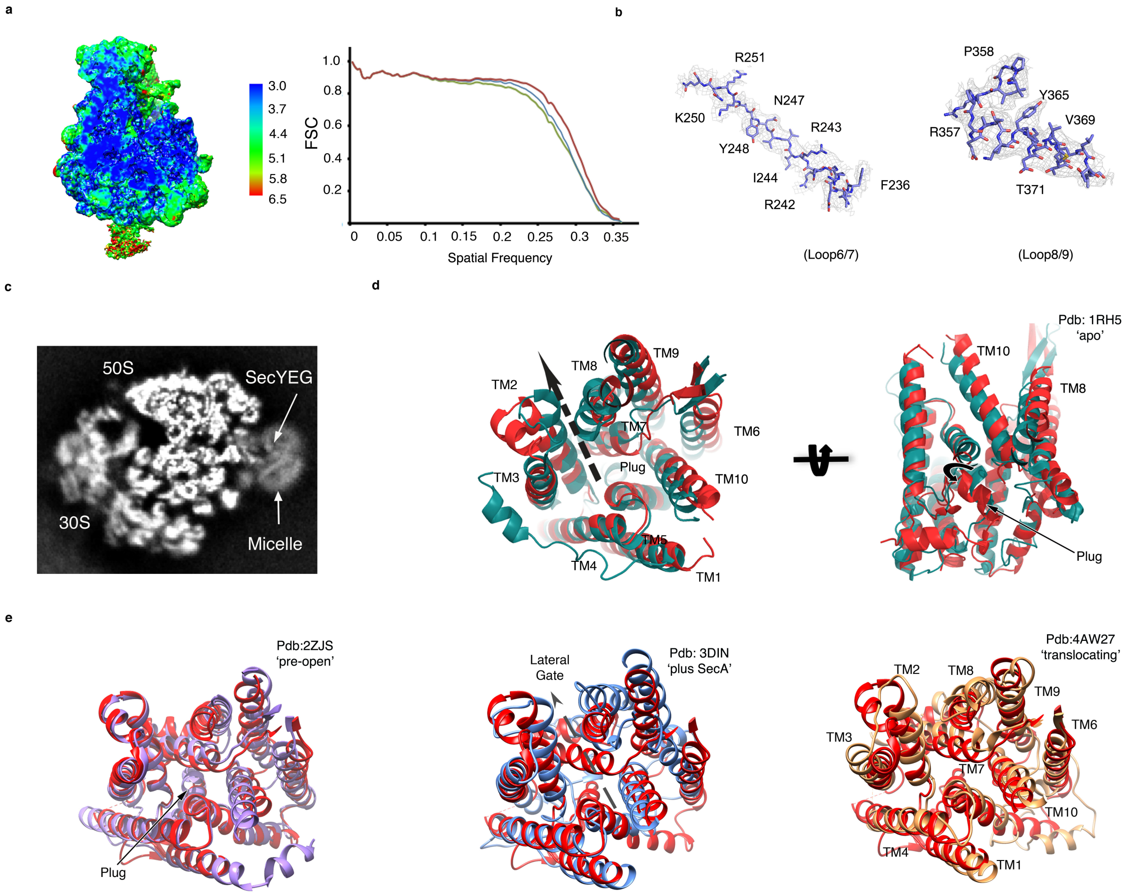

Supplementary Figure 10: Overview of the bacterial 'translocating' Sec translocon. **a**, Local resolution plot for the RNC-SecYEG targeting complex and refinement weight validation plot for the RNC-SRP refinement. FSC calculated for model versus map for half-dataset 1 (used for refinement) in blue, versus map for half-dataset 2 (not used for refinement) in green, versus full dataset in red. A larger gap for green versus blue plots would indicate over-refinement. **b**, Representative densities of SecY loop 6/7 and loop 8/9 with fitted built coordinates displayed as sticks. **c**, Cross-section of the unsharpened EM density map of RNC-SecYEG complex showing secondary structure elements of SecYEG within the unstructured micelle. **d**, Overlay of the archeal x-ray structure of the Sec-translocon in isolation (PDB:1RH5, green) and the bacterial RNC-bound translocon (red). Arrow indicates the movement of the plug away from the center of the channel. **e**, Superposition of the bacterial RNC-bound translocon (red) onto *T. thermophilus* in complex with Fab fragment (PDB:2ZJS, purple), the archeal translocon in complex with SecA (PDB:3DIN, blue) and the mammalian translocon in complex with a translating ribosome (PDB:4W27, orange).

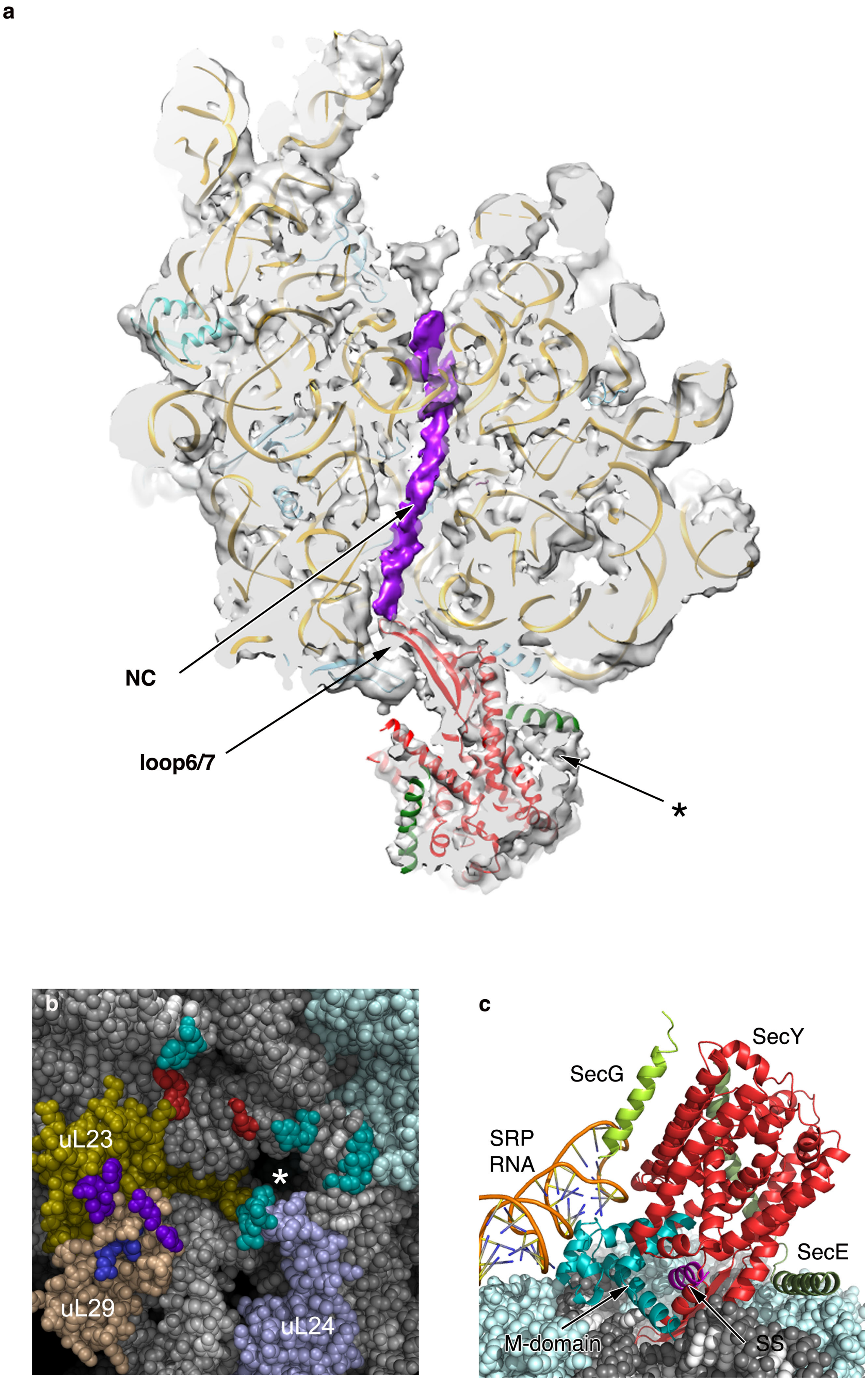
a
NC
loop6/7
*
b
uL23
*
uL29
uL24
c
SecY
SecG
SRP
RNA
SecE
M-domain
SS

Supplementary Figure 11: Co-binding mode of the SRP and Sec translocon on the RNC. a, Crossection of the RNC-SecYEG cryo-EM map (filtered to 4.8 Å resolution), with the EM-density for the SS sequence displayed in magenta. Asterisk indicates to the extra density at the lateral gate. **b**, Overview of the polypeptide exit site with the contact points of the targeting and membrane insertion factors. Contact points of the M domain, Ffh NG domain, the Sec translocon, and overlapping contact points with the ribosome are colored teal, blue, red, and purple, respectively. The white asterisk indicates the location of the ribosome tunnel. **c**, Composite atomic model of the SRP-SR-SecYEG complex. SecY, Sec E, and Sec G, were colored in red, dark green, and lime, respectively. M domain, SS, and SRP RNA are colored in turquoise, magenta, and orange, respectively. Ribosome coordinates are displayed as spheres.

Data collection, model refinement and validation	RNC-SRP-SR 'closed'	RNC-SRP Class 3	RNC-SecYEG
**Data collection**			
Particles	75,942	46,409	216,907
Pixel size (Å)	1.385	1.385	1.385
Defocus range (µm)	0.8-3.2	0.8-3.4	0.8-3.2
Voltage (kV)	300	300	300
Electron dose (e^{-}/A^{2})	20	20	20
**Reciprocal space data**			
Spacegroup	P1	P1	P1
$a = b = c$ (Å)	299.16	299.16	299.16
$\alpha = \beta = \gamma$ (°)	90.0	90.0	90.0
**Refinement**			
Resolution range (Å)	40.0 – 3.85	40.0 – 3.85	40.0 – 3.38
Applied geometry weight (wxc)	1.0	1.2	1.1
No. reflections	981,197	981,226	1,450,785
R-factor	0.226	0.233	0.235
No. residues			
Protein	3636	3636	3911
RNA	3049	3049	3007
Ligands (Mg^{2+}, Zn^{2+})	431/1	431/1	323/1
B-factors overall	86.2	50.8	73.7
Protein	94.9	60.3	84.7
RNA	82.7	47.0	68.7
Ligands (Mg^{2+},Zn^{2+})	47.3	11.8	32.1
R.m.s. deviations			
Bond lengths (Å)	0.007	0.007	0.008
Bond angles (°)	0.99	1.05	0.88
**Validation**			
Protein			
Molprobity clashscore	9.6	10.02	6.8
Ramachandran plot			
Favored (%)	96.9	96.6	96.8
Allowed (%)	3.0	3.3	3.1
Outliers (%)	0.1	0.1	0.1
RNA			
Correct sugar puckers (%)	99.4	99.4	99.5
Backbone outliers	0.0	0.0	0.0

Supplementary Table 1: Refinement table for the reciprocal space coordinate refinements of the *E. coli* RNC-SRP, 'closed' SRP-SR and RNC-SecYEG complexes

Supplementary References:

1. Larkin, M.A. et al. Clustal W and Clustal X version 2.0. *Bioinformatics* **23**, 2947-8 (2007).
2. Goujon, M. et al. A new bioinformatics analysis tools framework at EMBL-EBI. *Nucleic Acids Res* **38**, W695-9 (2010).
